# Treating incarcerated inguinal hernias with TEP is a viable option for experienced surgeons

**DOI:** 10.1038/s41598-020-77925-y

**Published:** 2020-11-30

**Authors:** Kayo Augusto de Almeida Medeiros, Bárbara Justo Carvalho, Leonardo Zumerkorn Pipek, Gustavo Heluani Antunes de Mesquita, Fernanda Nii, Diego Ramos Martines, Leandro Ryuchi Iuamoto, Luiz Augusto Carneiro-D’Albuquerque, Alberto Meyer, Wellington Andraus

**Affiliations:** 1 Faculdade de Medicina FMUSP, Universidade de São Paulo, São Paulo, Brazil; 2 Departamento de Gastroenterologia, Faculdade de Medicina, Hospital das Clínicas HCFMUSP, Universidade de São Paulo, Avenida Doutor Arnaldo, 455, São Paulo, Brazil; 3 Department of Orthopaedics and Traumatology, Center of Acupuncture, University of Sao Paulo School of Medicine, Sao Paulo, Brazil

**Keywords:** Anatomy, Gastroenterology

## Abstract

Despite inguinal hernias being a common problem in public health, there is still scarce information about the epidemiology of the complications, especially incarceration, and their influence on the laparoscopic surgical methods considering the role of the learning process of the surgeon. Compare laparoscopic totally extraperitoneal (TEP) approach in the repair of incarcerated and non-incarcerated inguinal hernias from the perspective of technical difficulty for trained surgeons. We obtained data about sex, age, location and type of hernia, surgery duration, ASA score, postoperative complications, previous surgeries and BMI. Groups were descriptively analyzed and statistically compared to verify how similar the samples were. 265 (90.1%) patients had non-incarcerated hernias and 29 (9.9%) incarcerated. We observed that there was no significant difference in the pattern of location (right, left or bilateral), sex, ASA, previous or complications between the two groups. Unilateral incarcerated hernias had longer operative times compared to non-incarcerated. No difference was found between bilateral hernias. We didn´t find significant epidemiological differences between incarcerated and non-incarcerated hernias. In our experience, with the limitation of a single-surgeon series, laparoscopic hernia repair achieved satisfactory results in terms of feasibility (especially for bilateral hernias) and safety.

## Introduction

Abdominal hernias are a common condition among the global population: it is estimated that around 20 million surgeries for hernia reapair are carried out annually. Among abdominal hernias, the inguinal hernia is the most prevalent^1^, constituting an important public health problem, especially when complications such as incarceration or strangulation occurs^2^.

Incarcerated hernia is defined as inability to manually reduce it back into the abdomen without signs of strangulation. With a risk of 2.8% after 3 months and 4.5% after 21 months^3^, this type of hernia can suffer ischemia and strangulation, increasing the risk of postoperative mortality from 0.01 to 5%^4^.

In view of these complications, the most appropriate method for repair of incarcerated inguinal hernias has been extensively discussed in the literature. The laparoscopic approach has been proved to be a feasible method for non-complicated hernias, with a low rate of clinical postoperative complications^5^, low length of postoperative hospital stay and less discomfort during recover when compared to open surgery. However, there is no significant difference in recurrence rates between the two approaches^6^. Due to the greater cost and technical difficulty^8^, the laparoscopic approach is the less common of the two, being used in the minority of procedures for hernia repair around the world^7^.

The aim of this study is to compare the laparoscopic totally extraperitoneal (TEP) approach in the repair of incarcerated and non-incarcerated inguinal hernias from the perspective of technical difficulty for trained surgeons. Therefore, being able to evaluate the technical feasibility of this procedure for this type of complication.

## Methods

This is a retrospective observational study of patients who have undergone TEP for the repair of incarcerated and non-incarcerated inguinal hernias, previously described by Iuamoto et al.^9^, Meyer et al.^7,10^ and Kassir et al.^11^. The procedures were carried out between May 2009 and July 2019 by a surgeon specialized in hernia repair in São Paulo, Brazil. A polypropylene mesh was used (weight 120 ± 10 g/m^2^, pores 0.9 ±  + / − 0.1 mm, size of 15 cm × 15 cm) per inguinal hernia^12^. The mesh was not trimmed for any patient.

Our inclusion criteria were patients older than 18 years who underwent elective repair of inguinal hernias by videolaparoscopy and who gave consent for the data use. This cohort consisted of 380 patients. Our exclusion criteria were the first 65 cases (for the overcome of the learning curve, according to the literature^9^) and cases operated by TAPP. 294 patients who were operated exclusively by TEP were included in our study.

We obtained data about sex, age, location and type of hernia, surgery duration, patient's overall health score from the American Society of Anesthesiology (ASA), postoperative complications, previous surgeries and body mass index (BMI) of patients.

Patients were followed for at least 1 one after the surgery. Complications were reassessed after 7, 15, 30, 182 and 365 days for all patients.

Qualitative variables will be expressed by absolute (n) and relative (%) frequency. Quantitative variables by mean, median, standard deviation, amplitude (minimum and maximum) and quartile range (1st quartile and 3rd quartile).

We will use the Student t-test or Mann–Whitney test to compare two groups of quantitative variables, depending on the assumption of data normality by the Kolmogorov–Smirnov test. To verify association in qualitative variables we will use Fisher's exact test. The adopted significance level was 5%.

The project was approved by the ethics committee of Samaritano Hospital, SP—Brazil.

### Compliance with ethical standards

All procedures performed in this study were in accordance with the ethical standards of the institutional research committee and with the 1964 Helsinki Declaration and its later amendments or comparable ethical standards.

### Informed consent

Informed consent was obtained from all individual adult participants included in this study.

## Results

Our analysis resulted in 294 patients, 265 (90.1%) with non-incarcerated hernias and 29 (9.9%) with incarcerated hernias. 288 patients (98%) were male. 155 (52.7%) cases were bilateral hernias. Patients with unilateral hernias were divided between 85 (28.9%) cases of hernias located on the right groin and 54 (18.4%) on the left. Only 9 (3.1%) patients were ASA 3, all others were ASA 1 or 2. 212 (72.1%) had no previous abdominal or pelvic surgeries. The mean age was 51.95 years old, mean BMI was 26.13 kg/m^2^ and mean operative time was 46.34 min (Table 1). 22.6% of non-incarcerated hernias were bigger than 2 cm. In the incarcerated group, 72.4% were bigger than 3 cm. Despite hernia size differences, the same mesh size was used for all patients.

**Table 1:** Comparison between incarcerated and non-incarcerated inguinal hernias on duration, BMI and age.

		Incarcerated (29)	Non-incarcerated (265)	p-value
Inguinal hernia (TOTAL)	Age (years)	﻿55.55 (﻿16.51)	﻿51.55 (﻿14.31)	0.218
	BMI	﻿﻿26.96 (﻿3.57)	﻿26.03 (﻿3.38)	0.189
	Surgery duration	﻿52.20 (﻿23.10)	﻿46.08 (﻿19.77)	0.179
Unilateral Inguinal hernia		Incarcerated (18)	Non-incarcerated (121)	
	Age (years)	﻿56.00 (19.23)	﻿51.01 (15.07)	0.305
	BMI	﻿26.88 (3.80)	﻿25.78 (3.42)	0.257
	Surgery duration	﻿50.44 (25.65)	﻿34.06 (14.12)	0.016*
Bilateral Inguinal hernia		Incarcerated (11)	Non-incarcerated (144)	
	Age (years)	﻿54.81 (11.54)	﻿56.18 (18.15)	0.456
	BMI	﻿27.09 (3.33)	﻿52.00 (13.68)	0.432
	Surgery duration	﻿55.09 (18.98)	﻿56.18 (18.15)	0.856

Data is presented as mean (SD) for continuous variables.

Complications occurred in just seven patients: six cases of scrotal hematoma and another of umbilical hematoma and edema (Clavien–Dindo I), all in non-incarcerated hernias. Recurrence occurred in two patients after 1 year with non-incarcerated hernia (2 men, ASA 2, with bilateral indirect hernias EHS: Lateral, 2). Linchtenstein conversion also occurred in two patients who had several adhesions after radical prostatectomy.

Comparing incarcerated and non-incarcerated hernias we observed that there was no significant difference in the pattern of location (right, left or bilateral), sex, ASA, previous surgeries or complications between the two groups. The most common previous surgery reported in both groups was unilateral hernia repair (Table 2).

**Table 2:** Demographic data comparison between incarcerated and non-incarcerated hernias.

Location	Incarcerated				Total		p-value
	No		Yes				
	N	%	N	%	N	%	
Left	47	17.70	7	24.10	54	18.40	0.2398
Right	74	27.90	11	37.90	85	28.90	
Bilateral	144	54.30	11	37.90	155	52.70	
**Sex**							
Male	260	98.10	28	96.60	288	98.00	0.4667
Female	5	1.90	1	3.40	6	2.00	
**ASA**							
1	114	43.00	9	31.00	123	41.80	0.4018
2	143	54.0	19	65.50	162	55.10	
3	8	3.00	1	3.40	9	3.10	
**Previous surgeries**							
No	191	72.10	21	72.40	212	72.10	0.6461
Yes	74	27.90	8	27.60	82	27.90	

Regarding surgery duration, incarcerated and non-incarcerated hernias were divided into two groups: unilateral and bilateral. Unilateral non-incarcerated hernias had a significant lower operative time with a mean duration of 34.06 min versus 50.44 min in the incarcerated group (p-value = 0.016) and there was no significant difference between these two groups in BMI or age (Table 1). Right hernias were more common in both incarcerated and non-incarcerated groups (61.1% × 61.2%, p = 1.0) and there was a higher frequency of indirect hernias (83.3%) in the incarcerated group than in the non-incarcerated (62%), p-value = 0.0286.

Bilateral hernias showed no significant difference in operating time, BMI or age (Table 1).

**Figure 1:**
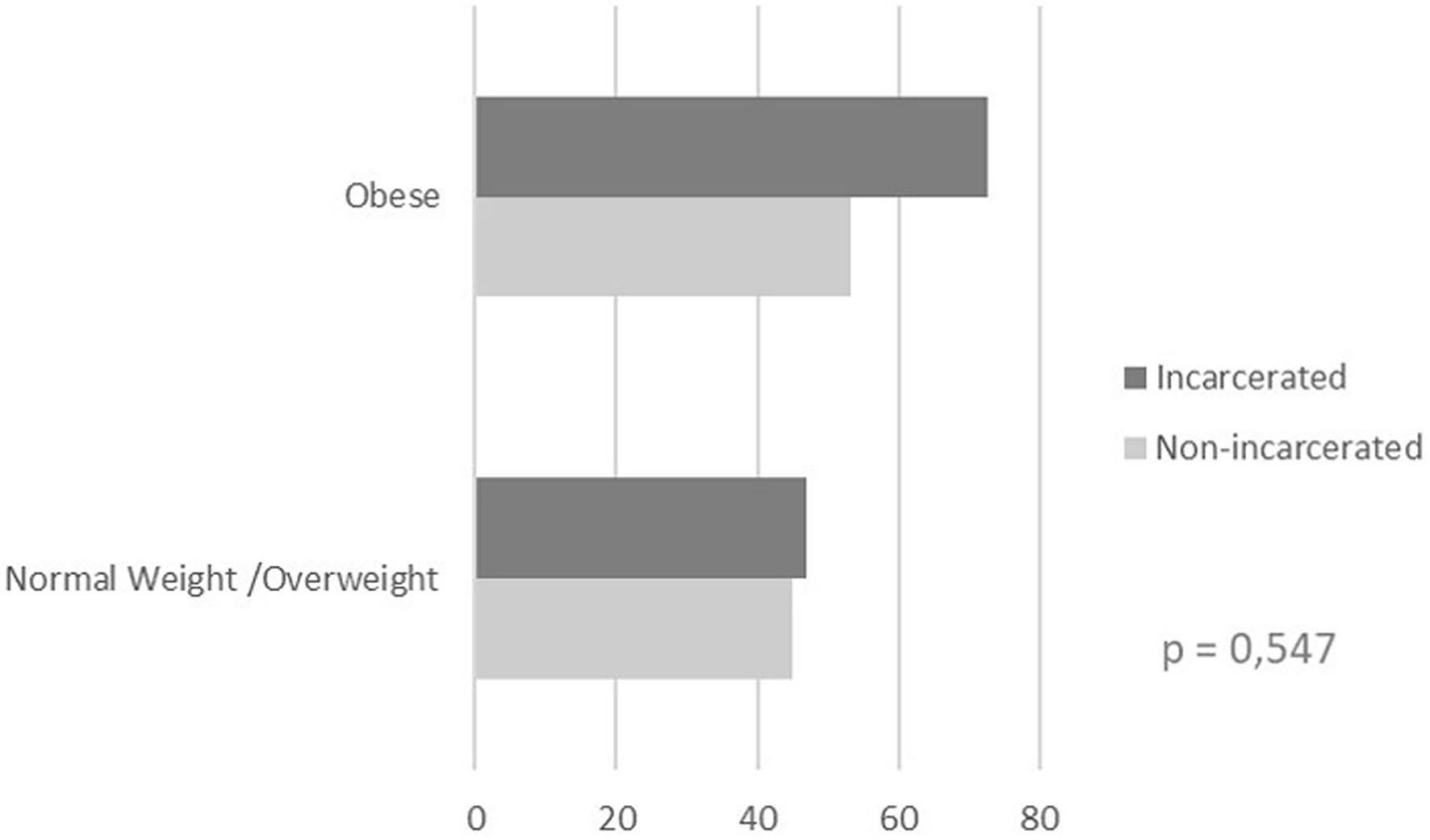
Mean operative time × BMI on incarcerated and non-incarcerated inguinal hernias. We considered as obese patients with BMI > 30 kg/m^2^.

When we analyzed influence of BMI in operative time, no difference could be observed between obese or non-obese patients with incarcerated and non-incarcerated hernias (Fig. 1).

## Discussion

There is no consensus in the literature on the best technique for operating on inguinal hernias. However, there are principles that guide the discussion; a good technique should present a low level of recurrence, allow the rapid recuperation of the patient with minimal morbidity, be easy to learn and to reproduce and be cost effective^13^.

Despite the laparoscopic technique still being little used worldwide (corresponding to just 16% in Denmark^14^ and less than 5% in Brazil^9^), many studies indicate it as a viable and effective method for repair of inguinal hernias.

With regard to incarceration and strangulation, the discussion is contradictory^4,6^, without widespread consensus and conclusions, even in guidelines^15^. Previous studies^16^ aimed to investigate the effectiveness of this approach in the repair of incarcerated/strangulated hernias by analyzing complication rate, recurrence, postoperative mortality and time to hospital discharge, but not from the perspective of the difficulty for trained surgeons in elective procedures. The number of patients in other previously published studies regarding the repair of incarcerated hernias for approaches other than the open repair ranged from 14 to 20, while we had 29 cases^17,18^.

Knowing that this minimally invasive surgery is a viable and effective procedure for non-incarcerated hernias, we sought to discover whether experienced surgeons were also able to operate incarcerated hernias with the same success. For this, we assessed the viability of extraperitoneal laparoscopic hernioplasty for the repair of incarcerated inguinal hernias using operating time as the key criteria to compare incarcerated and non-incarcerated hernias.

Before the main analysis, the two groups (incarcerated and non-incarcerated) were compared to exclude differences in the epidemiological profiles that could interfere in the analysis of the operating times. In this comparison, there was no significant difference in the pattern of location (right, left or bilateral), sex, ASA, previous surgeries or complications between the two groups. The groups were not statistically different in terms of epidemiological categories, a similar result to that found in the study of Wakasugui et al.^17^.

The duration of surgery performed by experienced surgeons presented in the international literature are similar to those found in our study^16,19^. Wakasugui et al.^17^ found a significant difference in operating time between unilateral incarcerated hernias and the control group, composed of non-incarcerated unilateral hernias (111 min vs 76 min). In our analysis also there was a significant difference between these groups.

In the analysis of bilateral hernias, Wakasugui et al. did not find statistically significant differences in operating time between incarcerated and non-incarcerated hernias, similar to our results.

It is possible to elaborate some possible explanations for this similarity between our work and the literature. The time required in operating a unilateral incarcerated hernia is proportionally shorter than in bilateral incarcerated hernias, as only one side is incarcerated. Another possible explanation is that the surgeon only operates bilateral incarcerated cases after mastering the technique in unilateral cases.

Willoughby et al.^20^ found that patients with BMI > 30 and unilateral inguinal hernias submitted to laparoscopic repair had fewer complications compared to open surgery, thus there was no impediment to laparoscopic surgery, despite longer operative times as BMI increased. In our study, the increase in BMI was also responsible for an increase in operative time but did not significantly increase the operative difficulty of incarcerated in relation to non-incarcerated hernias, as seen by the no difference in operative time between groups.

Moreover, postoperative pain is an important variable when deciding the best surgical technique. In TAPP procedures^21^, inguinal hernia repair with glue fixation significantly decreased the frequency and intensity of the pain when compared with the pain before the surgery, showing the benefits of hernia correction for pain management. Asuri et al.^22,23^ showed that TEP was superior than TAPP with reduced postoperative pain up to 3 months reported by patients, but no difference in overall mortality or complications. Moreover, surgical fixation in TEP procedures has been shown not significant for postoperative pain^24^. The use of Infiltration of bupivacaine into the preperitoneal space and trocar incisions is a promising technique to reduce postoperative pain^25^.

Besides the surgical technique, many factors are important for pain and mortality in hernia repair. Mitura et al.^26^ observed that the occurrence and intensity of pain was significantly higher in younger patients. Other characteristics may play a role, not only in pain, but also morbidity and mortality, this include smoking^27^, previous intestinal resection^28^ and concomitant diseases^28,29^.

Among the limitations of this study are the small number of cases and the fact that it is based only on a series of procedures carried out by one surgeon. A multicenter randomized study would allow for broader conclusions.

## Conclusions

We did not find significant epidemiological differences between incarcerated and non-incarcerated hernias. In our experience, with the limitation of a single-surgeon series, laparoscopic hernia repair achieved satisfactory results in terms of feasibility (especially for bilateral hernias) and safety. Few series about this topic were published. More prospective trials are needed.
